# How does the side‐effect information in patient information leaflets influence peoples’ side‐effect expectations? A cross‐sectional national survey of 18‐ to 65‐year‐olds in England

**DOI:** 10.1111/hex.12584

**Published:** 2017-06-15

**Authors:** Rebecca K. Webster, John Weinman, G. James Rubin

**Affiliations:** ^1^ NIHR Health Protection Research Unit in Emergency Preparedness and Response King's College London London UK; ^2^ Department of Psychological Medicine Institute of Psychiatry, Psychology & Neuroscience King's College London London UK; ^3^ Institute of Pharmaceutical Science King's College London London UK

**Keywords:** descriptors, expectations, patient information leaflets, risk, side effects

## Abstract

**Objectives:**

To establish how the terms recommended by the European Commission to describe side‐effect risk in patient information leaflets (PILs) influences expectations of side‐effects and to identify factors associated with these side‐effect expectations.

**Design:**

A cross‐sectional online survey was carried out by a market research company.

**Setting:**

Data were collected in England between 18th March and 1st April 2016.

**Participants:**

A total of 1003 adults aged between 18 and 65. Main outcome measures: Self‐reported expectation that the described side‐effects would affect participants if they took the medicine, measured on a likelihood scale from 1 (very unlikely) to 5 (very likely).

**Results:**

Participants formed high expectations of side‐effects for “very common” and “common” side‐effects, with 51.9% and 45.0% of participants rating these as “very likely” or “likely” to happen to them, respectively. This fell to 8.1% for “uncommon,” 5.8% for “rare” and 4.1% for “very rare.” For each descriptor, higher expectations of side‐effects were more associated with women or being from an ethnic minority, or having less education, a household illness, high perceived sensitivity to medicines or negative beliefs about medicines.

**Discussion:**

The current use of verbal descriptors to communicate side‐effect risk in PILs leads to high side‐effect expectations. These expectations could contribute to nocebo‐induced medication side‐effects experienced by patients. Additional work is required to identify ways to improve the way risk information is conveyed in PILs.

## 1 INTRODUCTION

Medications may generate adverse reactions, with studies showing that around 6.5% of patient admissions to hospitals are related to an adverse drug reaction (ADR).^1^ ADRs are noxious, unintended responses to medication which occur at normal doses.^2^ Medication side‐effects such as these can significantly reduce patient adherence^3^ often leading to reduced therapeutic benefit for the patient. They also mean financial costs for health services as they are a major cause of hospital admissions, and emergency department and outpatient care visits.^4^

Not all side‐effects are related to the physiological action of the medication, however.^5^ For example, it has been noted that only 10.9% of adverse reactions reported to common prescription drugs are clearly attributable to the medication.^6^ Many other, non‐specific, side‐effects may occur due to a nocebo effect.^7^ Nocebo effects have been described as the flip side to the placebo effect, whereby an adverse reaction is experienced by someone who receives an inert exposure.^8^ From a recent systematic review, we know that expectations such as those generated from verbal suggestions of what symptoms to expect are one of the strongest factors affecting the development of nocebo effects.^9^ Expectations have also been found to contribute to the side‐effects that patients experience from their medications.^10^, ^11^ This is a problem as side‐effects are an important cause of patient non‐adherence.^3^, ^12^

One of the main ways that patients can generate these negative expectations of their medication is by reading the accompanying patient information leaflet (PIL). Over 70% of patients will read the accompanying PIL for a newly prescribed medication.^13^ In Europe, all medicines prescribed or sold over the counter must be distributed with a comprehensive PIL.^14^ In 1998, European Commission (EC) guidelines advised PILs should group side‐effects according to five frequency bands, using a different verbal label for each one.^15^ As such, side‐effects could be grouped into “very common” (affect more than 1 in 10 patients), “common” (up to 1 in 10), “uncommon” (up to 1 in 100), “rare” (up to 1 in 1000) or “very rare” (up to 1 in 10 000).

However, as the guidelines were published, several studies have shown that these verbal labels are problematic, leading to overestimations by samples of students, patients and health‐care professionals.^16^, ^17^, ^18^, ^19^, ^20^, ^21^, ^22^, ^23^ As such, current guidelines suggest combining verbal and frequency expressions (eg, “very common, more than 1 in 10 people”).^24^ Although it has been shown that this may not lead to more accurate side‐effect risk estimates than the verbal format^25^ and still leads to significant risk overestimations when compared to numerical frequency bands alone.^26^ In part, patient estimations seem to depend on the type of side‐effect with “mild” side‐effects generally resulting in higher estimations than “severe” side‐effects.^19^

Although previous studies have looked at how these verbal descriptors affect people's numerical estimation of side‐effects, they have not looked at how they affect people's subsequent expectations of side‐effects. The two issues are linked, but not identical. It is possible for an optimistic patient to believe that symptoms are common but unlikely to affect them personally, and vice versa. This is important as it is these expectations that may trigger a nocebo response. This study investigated people's expectations of side‐effects when described using EC recommended descriptors. We also tested whether these expectations are associated with demographic and psychological characteristics such as beliefs about medicines, optimism or perceived sensitivity to medicines which have previously been implicated in the nocebo literature.^5^, ^27^

Our specific aims were to

1. Assess people's expectations of side‐effects from the EC recommended risk descriptors
2. Investigate if these expectations differ depending on whether they relate to mild or severe side‐effects.
3. Determine whether demographic factors (age, gender, ethnicity, employment status, level of education or presence of a household illness) are associated with the expectation of experiencing a side‐effect after taking a medicine labelled with one of the EC recommended risk descriptors.
4. Determine whether psychological factors (optimism, perceived sensitivity to medicines, belief about medicines, health anxiety, health literacy or PIL reading behaviour) are associated with the expectation of experiencing a side‐effect after taking a medicine labelled with one of the EC recommended risk descriptors.
5. Determine if participants’ understanding of what the verbal descriptors mean is associated with their subsequent expectations of side‐effects.

## 2 METHODS

### 2.1 Design

The market research company Ipsos MORI conducted an online survey of adults aged between 18 and 65 living in England on our behalf, between 18th March and 1st April 2016. This study was approved by the Psychiatry, Nursing and Midwifery Research Ethics Committee at King's College London (ref: HR‐15/16‐2104).

The same study was used to assess in detail factors associated with how patients understand the numerical risk information conveyed by verbal labels of risk, the results of which have been submitted elsewhere.

### 2.2 Participants

Participants were recruited by Ipsos MORI using their existing database of people living in England and interested in taking part in Internet surveys (approximate n=160 000). We excluded over 65s because of concerns about the representativeness of this group in Internet surveys.^28^, ^29^ Potential participants were emailed a link to the survey. After providing informed consent, participants were allocated by the survey software to receive questions about either mild or severe side‐effects. The allocation was based on which condition had the lowest number of completed responses at that time. Panel participants received points for completing the survey equivalent to 75 pence.

### 2.3 Sample size

We used quotas based on the National Readership Survey^30^ to ensure that the sample reflected the demographic profile of 18‐ to 65‐year‐olds in England. This is standard method for this form of research to ensure samples are representative of the adult English population. These quotas were based on participant age and gender (interlocked), location and working status. A priori we intended to recruit 1000 participants to provide us with a sample error of about plus or minus 3%.

### 2.4 Questionnaire development

Where possible, we included or adapted items that had been previously developed and tested for their reliability and validity and that have been widely used in the literature. Using opportunity sampling, we piloted all items with five members of the general public who read through the questionnaire with the researcher and identified anything that was not clear. We rephrased items where necessary to improve clarity. See supporting information for full copy of the questionnaire with top line results.

### 2.5 Primary outcome: Side‐effect expectations

Five items were used to assess participant expectations of side‐effects. Depending on which condition they had been assigned to, participants were told that a new drug had been developed with either “dizziness” or “kidney failure” as one of its side‐effects. The side‐effect was described using each of the five EC recommended combined verbal and frequency expressions (eg, very common, may affect more than 1 in 10 people) which were presented in a random order. Participants were asked to rate how likely they were to experience dizziness or kidney failure if they took the drug on a five point scale ranging from “very unlikely to very likely.”

### 2.6 Demographic factors

Participants were asked about their age, gender, ethnicity, highest level of education, employment status and whether they or anyone in their household had a long‐standing illness, disability or infirmity.

### 2.7 Psychological factors

A single item to assess health literacy was adapted from elsewhere^31^ and asked participants to state how often they needed help reading PILs. We also included one question which asked how often participants read PILs when taking a new medication. Response options for both ranged from 1 (“never”) to 5 (“always”). We assessed health anxiety using one question from the health anxiety inventory.^32^ This asked participants to rate themselves from 1 “I do not worry about my health” to 4 “I spend most of my time worrying about my health.”

The Revised Life Orientation Test^33^ was used to rate participant optimism. This has six questions and provides a score from 6 (least optimism) to 30 (most optimism). We used the overuse and harm general subscales from the Beliefs about Medicines Questionnaire (BMQ)^34^ to measure attitudes towards medicines in general. These subscales give scores from 4 to 20, with higher scores indicating higher perceived overuse or harm. The Perceived Sensitivity to Medicines scale^35^ was used to assess how sensitive participants thought they were to medicines. This provides a score from 5 to 25 with higher scores indicating higher sensitivity.

### 2.8 Participant understanding of verbal risk descriptors

We included five items (presented in a random order) to assess participant understanding of the side‐effect risk descriptors (“very common,” “common,” “uncommon,” “rare” and “very rare”). These asked people to consider a PIL for an imaginary drug which stated, for example, that “nausea is common.” Participants were asked to estimate how many out of 10 000 people who take the drug would develop that side‐effect. Participants were asked about either mild side‐effects (“headache” or “nausea”) or severe side‐effects (“seizure” or “difficulty breathing”) depending on which condition they had been assigned to.

### 2.9 Analysis

Participants’ expectations were grouped by likelihood to see the frequency that each likelihood statement was selected for the different risk descriptors. We carried out a series of chi‐squared tests to see whether participant expectations differed between mild and severe side‐effects. For occurrences where the expected cell count was below 5, Fisher's exact test was used instead.

Ordinal regressions were carried out to identify if any demographic or psychological characteristics, or how well participants estimated the EC recommended descriptors, were associated with expectations of personally experiencing side‐effects. The dependent variable for each regression was participants’ scores on the likelihood scale for each verbal descriptor. For each regression, all demographic variables and side‐effect type (mild or severe) were added in one block, and each psychological variable was added on its own, controlling for the previously entered variables.

For all analyses, answers of “don't know” or “prefer not to say” were excluded. Only 3% of participants answered “don't know,” and 1% answered “prefer not to say” for any question where this was an option. Analyses were carried out using SPSS 22. As participant expectations did not change by more than 0.2% when using data weighted by age, gender, region and working status, our analyses used unweighted data.

## 3 RESULTS

### 3.1 Sample characteristics

**Figure 1:**
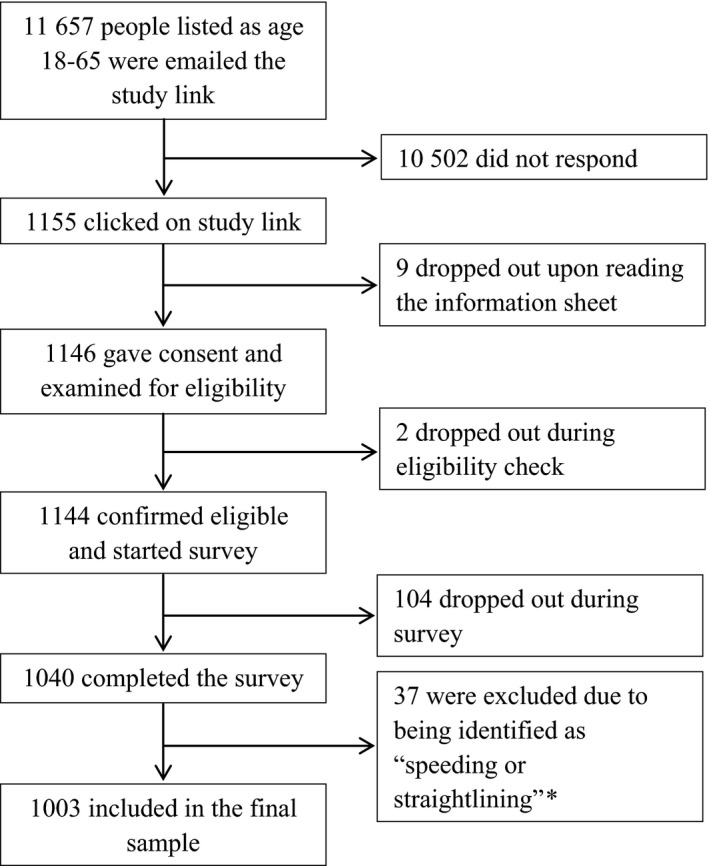
Participant flow through the survey. Eligibility check was used to confirm participants’ age in case this had changed since they were emailed the link. * Completing the survey too quickly to have given genuine, considered answers, or providing identical answers to five or more consecutive questions where this was possible.

A total of 1003 participants completed the survey and were included in the final sample (see Figure 1 for response rates). Demographic information for the participants is given in Table 2.

### 3.2 The influence of the EC recommended combined risk descriptors on participant expectations

Table 1 shows participant expectations of side‐effects for each of the descriptors. Expectations of side‐effects varied widely for each descriptor; however, the majority of participants thought that “very common” and “common” side‐effects were very likely or likely to happen to them (51.9% and 45%, respectively) despite these terms only being intended to represent a risk of around 1 in 10 patients being affected. Expectations of side‐effects dropped substantially for “uncommon,” “rare” and “very rare” with 8.1%, 5.8% and 4.1% of participants thinking there were very likely or likely to experience the side‐effects, respectively.

**Table 1:** Expected likelihood of minor (dizziness) and severe (kidney failure) side‐effects from an imagined new drug using the EC recommended combined descriptors

Current guidelines	Expectancy	Mild, n (%)	Severe, n (%)	Total, n (%)
Very common (more than 1 in 10)	Very likely	115 (22.7)	106 (21.3)	221 (22.0)
	Likely	152 (30.0)	148 (29.8)	300 (29.9)
	About as likely as not	118 (23.3)	128 (25.8)	246 (24.6)
	Unlikely	91 (18.0)	79 (15.9)	170 (16.9)
	Very unlikely	20 (4.0)	20 (4.0)	40 (4.0)
	Don't know	10 (2.0)	16 (3.2)	26 (2.6)
	Chi‐square test	χ^2^=1.443, *P*=.837		
Common (up to 1 in 10)	Very likely	58 (11.5)	63 (12.7)	121 (12.1)
	Likely	175 (34.6)	155 (31.2)	330 (32.9)
	About as likely as not	122 (24.1)	132 (26.6)	254 (25.3)
	Unlikely	121 (23.9)	114 (22.9)	235 (23.4)
	Very unlikely	20 (4.0)	19 (3.8)	39 (3.9)
	Don't know	10 (2.0)	14 (2.8)	24 (2.4)
	Chi‐square test	χ^2^=1.874, *P*=.759		
Uncommon (up to 1 in 100)	Very likely	7 (1.4)	10 (2.0)	17 (1.7)
	Likely	32 (6.3)	32 (6.4)	64 (6.4)
	About as likely as not	92 (18.2)	103 (20.7)	195 (19.4)
	Unlikely	215 (42.5)	217 (43.7)	432 (43.1)
	Very unlikely	154 (30.4)	121 (24.3)	275 (27.4)
	Don't know	6 (1.2)	14 (2.8)	20 (2.0)
	Chi‐square test	χ^2^=4.827, *P*=.306		
Rare (up to 1 in 1000)	Very likely	3 (0.6)	7 (1.4)	10 (1.0)
	Likely	28 (5.5)	20 (4.0)	48 (4.8)
	About as likely as not	56 (11.1)	72 (14.5)	128 (12.8)
	Unlikely	136 (26.9)	169 (34.0)	305 (30.4)
	Very unlikely	278 (54.9)	218 (43.9)	496 (49.5)
	Don't know	5 (1.0)	11 (2.2)	16 (1.6)
	Chi‐square test	a=15.452, *P*=.003		
Very Rare (up to 1 in 10 000)	Very likely	5 (1.0)	6 (1.2)	11 (1.1)
	Likely	15 (3.0)	15 (3.0)	30 (3.0)
	About as likely as not	38 (7.5)	51 (10.3)	89 (8.9)
	Unlikely	65 (12.8)	70 (14.1)	135 (13.5)
	Very unlikely	375 (74.1)	342 (68.8)	717 (71.5)
	Don't know	8 (1.6)	13 (2.6)	21 (2.1)
	Chi‐square test	χ^2^=3.495, *P*=.481		

Chi‐square analyses did not include participants who answered “Don't know”.

a One cell had an expected cell count of less than 5 so Fisher's exact was used instead.

### 3.3 Does the severity of the side‐effect affect participant expectations?

The difference in expectations between mild and severe side‐effects is shown in Table 1. There was no difference in expected likelihood between mild and severe side‐effects except for side‐effects described as “rare” in which expectations were lower for mild vs severe side‐effects, *P*=.003.

### 3.4 Are demographic factors associated with expectations?

Table 2 shows the association between demographic variables and perceived likelihood of experiencing side‐effects, with all demographic variables controlled for each other. Men were 31% less likely than women to have higher expectations of side‐effects described as being “very common” or “common.” Participants from ethnic minorities were more likely to have higher expectation of side‐effects being as “common, uncommon, rare and very rare.” Compared to those with a university degree, participants with no academic qualifications were more likely to have a higher expectation of side‐effects described as “uncommon, rare and very rare.” Similarly compared to having a university degree, participants with school qualifications were more likely to have a higher expectation of side‐effects described as “uncommon, rare and very rare.” Having an illness or an illness in the household increased expectations of side‐effects compared to having no household illness but this was only significant for some of the descriptors (“very common,” “common” and “uncommon”).

**Table 2:** Demographic and psychological factors associated with participant expectations that very common, common, uncommon, rare and very rare side‐effects would personally affect them

Variable	No (%) or median (IQR)	Very common Adjusted odd ratios (95% CI) n=977	Common Adjusted odd ratios (95% CI) n=979	Uncommon Adjusted odd ratios (95% CI) n=983	Rare Adjusted odd ratios (95% CI) n=987	Very rare Adjusted odd ratios (95% CI) n=982
Gender^a^						
Male	492 (49.1%)	**0.69 (0.46‐0.92)**	**0.69 (0.55‐0.87)**	1.05 (0.83‐1.33)	0.88 (0.69‐1.12)	0.94 (0.70‐1.25)
Female	511 (50.9%)	Reference	Reference	Reference	Reference	Reference
Age^a^	41.0 (22.0)	0.99 (0.98‐1.00)	1.00 (0.99‐1.01)	0.99 (0.98‐1.002)	1.00 (0.99‐1.01)	1.00 (0.99‐1.01)
Ethnicity^a^						
Ethnic minorities	107 (10.7%)	1.25 (0.86‐1.83)	**1.49 (1.02‐2.18)**	**2.09 (1.42‐3.07)**	**2.06 (1.41‐3.03)**	**2.33 (1.53‐3.56)**
White	886 (88.3%)	Reference	Reference	Reference	Reference	Reference
Employment^a^						
Not working	280 (27.9%)	1.07 (0.81‐1.39)	1.01 (0.78‐1.32)	1.02 (0.78‐1.33)	0.90 (0.68‐1.19)	0.79 (0.56‐1.10)
Working	723 (72.1%)	Reference	Reference	Reference	Reference	Reference
Education^a^						
No academic qualifications	44 (4.4%)	0.82 (0.47‐1.46)	1.23 (0.69‐2.20)	**3.06 (1.70‐5.51)**	**2.73 (1.52‐4.92)**	**4.88 (2.62‐9.06)**
School qualifications	387 (38.6%)	0.94 (0.74‐1.19)	0.99 (0.77‐1.25)	**1.42 (1.11‐1.81)**	**1.52 (1.18‐1.96)**	**1.55 (1.14‐2.10)**
University degree	565 (56.3%)	Reference	Reference	Reference	Reference	Reference
Household illness^a^						
Yes—me	290 (28.9%)	1.27 (0.97‐1.66)	**1.40 (1.07‐1.83)**	**1.42 (1.07‐1.87)**	1.13 (0.85‐1.50)	1.23 (0.88‐1.71)
Yes—someone else	128 (12.9%)	**1.47 (1.04‐2.10)**	**1.78 (1.25‐2.54)**	**1.85 (1.29‐2.65)**	1.17 (0.81‐1.70)	1.06 (0.68‐1.65)
No	571 (56.9%)	Reference	Reference	Reference	Reference	Reference
Side‐effect type^a^						
Mild	506 (50.4%)	1.03 (0.82‐1.29)	1.00 (0.80‐1.26)	0.80 (0.63‐1.12)	**0.70 (0.55‐0.89)**	0.77 (0.58‐1.03)
Severe	497 (49.6%)	Reference	Reference	Reference	Reference	Reference
Estimates 1^b^						
Incorrect (under for Very common, over for Very rare)	‐	0.83 (0.63‐1.10)	‐	‐	‐	**0.55 (0.37‐0.83)**
Correct	‐	Reference	‐	‐	‐	Reference
Estimates 2^b^						
Under	‐	‐	0.94 (0.60‐1.48)	**1.54 (1.10‐2.16)**	1.29 (0.79‐2.09)	‐
Over	‐	‐	0.96 (0.70‐1.33)	1.25 (0.95‐1.64)	1.08 (0.80‐1.46)	‐
Correct	‐	‐	Reference	Reference	Reference	‐
Optimism^b^	19.0 (6.0)	0.98 (0.95‐1.00)	0.98 (0.96‐1.01)	0.97 (0.95‐1.001)	0.98 (0.95‐1.00)	0.98 (0.95‐1.02)
Perceived sensitivity to medicines^b^	10.0 (6.0)	**1.05 (1.02‐1.08)**	**1.05 (1.02‐1.08)**	**1.07 (1.04‐1.10)**	**1.10 (1.07‐1.14)**	**1.10 (1.07‐1.14)**
BMQ overuse^b^	12.0 (4.0)	1.04 (1.00‐1.08)	**1.05 (1.01‐1.09)**	**1.08 (1.04‐1.12)**	**1.05 (1.01‐1.09)**	**1.08 (1.03‐1.14)**
BMQ harm^b^	10.0 (4.0)	1.04 (1.00‐1.08)	**1.07 (1.03‐1.12)**	**1.14 (1.10‐1.19)**	**1.17 (1.12‐1.22)**	**1.20 (1.13‐1.26)**
Healthy anxiety^b^	2.0 (0.0)	**1.25 (1.02‐1.53)**	1.13 (0.93‐1.39)	1.14 (0.93‐1.40)	0.98 (0.79‐1.20)	1.07 (0.84‐1.37)
Health illiteracy^b^	1.0 (1.0)	1.06 (0.94‐1.20)	**1.14 (1.004‐1.29)**	**1.44 (1.27‐1.63)**	**1.49 (1.31‐1.69)**	**1.47 (1.28‐1.68)**
PIL reading frequency^b^	4.0 (2.0)	1.00 (0.91‐1.11)	0.97 (0.87‐1.07)	0.96 (0.86‐1.06)	0.96 (0.86‐1.07)	**0.88 (0.78‐0.999)**

^a^Controlled for each other, ^b^controlled for variables^a^.

IQR, interquartile range; CI, confidence interval; BMQ, Belief about Medicines; PIL, patient information leaflet; **bold=** *P*<.05, n ≠1003 due to excluding “don't know” responses.

### 3.5 Are psychological factors associated with expectations?

Table 2 shows the association between psychological variables and perceived likelihood of experiencing side‐effects, controlling for demographic characteristics. People with a higher perceived sensitivity to medicines were 5%‐10% more likely to have higher expectations of side‐effects described using each of the descriptors compared to those with a lower perceived sensitivity to medicines. Participants who thought medicines were overused or caused harm were 5%‐10%, and 7%‐20%, respectively, more likely to have higher expectations of side‐effects described as “common,” “uncommon,” “rare” or “very rare.” Participants scoring higher on health anxiety were 25% more likely to have higher expectations of side‐effects described as “very common” compared to those with lower health anxiety. Participants who needed help reading PILs were 14%‐49% more likely to have higher expectations of side‐effects described as “common,” “uncommon,” “rare” and “very rare.” Participants who read PILs more often were 12% less likely to have higher expectations of side‐effects described as “very rare” compared to those who read PILs less often. There was no effect of optimism on participants’ side‐effect expectations.

### 3.6 Are participants’ numerical estimates of the risk descriptors associated with their subsequent side‐effect expectations?

Whether participants estimated each of the EC recommended descriptors in accordance with the corresponding EC frequency band had little effect on their side‐effect expectations, apart from the descriptor “very rare” and “uncommon.” Participants who overestimated the number of patients likely to experience a “very rare” side‐effect were 45% less likely to have higher expectations of “very rare” side‐effects, and participants who underestimated the intended meaning of “uncommon” were 54% more likely to have higher expectations of uncommon side‐effects. Full results of participants’ estimations have been submitted elsewhere.

## 4 DISCUSSION

### 4.1 Summary of main findings and interpretation

There are several key findings from our work. First, when presented with the standard format of side‐effect risk information that is currently used in PILs, people form high expectations about their personal likelihood of experiencing symptoms, with the majority of people thinking “very common” or “common” side‐effects are likely to happen to them, despite those descriptors only representing a risk of around 1 in 10 people being affected. However, these expectations are formed independently from probability estimates. Under, over or correctly estimating the numerical meaning of a side‐effect risk descriptor had little bearing on whether a patient felt that they, personally, would experience it. These high side‐effect expectations are problematic, as they can be an important precursor to the development of actual side‐effects, as a result of a nocebo effect.^9^ Although reading about side‐effects does not always cause someone to experience a side‐effect,^36^, ^37^ in some situations it can.^10^, ^11^ Therefore, it is important to reduce any unrealistically high side‐effect expectations that PILs produce.

Second, similar to the nocebo literature,^9^ participants’ expectation of side‐effects described using the current guidelines does show associations with demographic and psychological factors. This will allow clinicians to be aware of those patients more at risk of developing nocebo‐induced side‐effects to their medications as a result of high side‐effect expectations. Women are more likely to expect higher risk side‐effects compared to Men, and people from ethnic minorities are more likely to expect side‐effects than people who are White. This supports previous research that has shown women and people from ethnic minorities have more dread of potential risks/hazards in general.^38^ Participants with no academic qualifications and those with school qualifications are more likely to expect lower risk side‐effects than those with university degrees. This may be because these participants are less familiar with and have difficulty interpreting the terms “1 in 1000” and “1 in 10 000” that accompanied the verbal descriptors in these questions and therefore misinterpret what it means in terms of their personal side‐effect expectations. Similarly higher health illiteracy was associated with higher expectations of side‐effects. Having a household illness increased expectations of side‐effects compared to not having a household illness, possibly due to an availability heuristic^39^ as symptoms are more likely to be present if there is a household illness. Interestingly, despite being implicated in the literature on people's numerical estimates of the descriptors,^19^ we found side‐effect type had little effect on participants’ expectations. Similarly whether participants provided correct or incorrect risk estimates for the descriptors had little effect on expectations. There are two possible explanations for this. First, the verbal risk descriptors do not influence expectations. Or second, the inclusion of the numerical expression draws people's attention away from the verbal risk descriptor. Either way the verbal risk descriptors are ineffective at influencing people's expectations. These support the view that participants form independent expectations of side‐effects from any generalized, numerical risk estimates.

In terms of the psychological factors, perceived sensitivity to medicines showed the strongest association with expectation; participants with a higher perceived sensitivity were more likely to expect side‐effects than those with a lower perceived sensitivity. In addition, a stronger belief that medicines are overused and cause harm increased the expected likelihood of side‐effects. This supports previous research showing negative views about medicines translate into negative expectations.^5^ Perceived sensitivity to medicines and negative belief about medicines have also been found to determine side‐effect reporting to vaccinations and new medication.^40^, ^41^ It is likely that the relationship between these factors and symptom reporting is mediated by negative expectations. It may be important to combat these negative medication beliefs in the first instance to reduce patients experiencing nocebo‐induced side‐effects as a result of negative expectations.

### 4.2 Implications for side‐effect reporting guidelines and clinical practice

Verbal descriptors have long been favoured for the presentation of side‐effect risk information.^42^ However, previous research including our own large‐scale survey (submitted elsewhere) has shown that verbal descriptors mislead rather than inform, leading readers to greatly overestimate the risk of side‐effects. Given that our analyses in this study suggest that participant estimations of the currently used descriptors also have little if any impact on patient expectations of their own likelihood of experiencing side‐effects, the rationale for using them appears to be weak. As well as having implications for PILs, our results also highlight the need for clinicians to assess patients’ understanding of the side‐effect information and the risk of side‐effects occurring, before explaining the likelihood of side‐effects based on information in PILs and providing reassurance to patients if necessary. This is particularly important for patients with risk factors for overestimating their likelihood of developing side‐effects, namely women, those with lower educational attainment, those with a household illness, those who seem to have difficulty reading health‐related literature, those with a perceived sensitivity to medicine and negative beliefs about the overuse and harm of medicine.

### 4.3 Strength and weaknesses, and future research

This study included a large sample size of 18‐ to 65‐year‐olds in the English population, specifically recruited to demographically reflect the composition of the English population on multiple key variables (age, gender, location and working status). Whether members of market research panels are psychologically representative of the general population in terms of attitudes to medicines and their expectations of side‐effects are unknown, however. In addition, because we were interested in participants’ perceptions of verbal descriptors rather than side‐effects and also to avoid confusing participants when they came to answer our primary outcome questions we chose to use different side‐effects to assess participants’ understanding of the descriptors than those used in the primary side‐effect expectations outcome. It is plausible that had we used the same symptom in the estimation measure we would have identified stronger associations with our primary outcome.

Many questions used in the survey were hypothetical, asking participants to state their expectations of side‐effects for an imaginary drug, for example. Future research could usefully build on this study by testing whether the findings hold true for patients given a newly prescribed medication. Due to the hypothetical nature, despite research showing there is some evidence that previous experience influences nocebo effects,^9^ we could not assess participants’ previous experience with this imaginary drug. We did, however, include the perceived sensitivity to medicines measure which assessed participants’ past reactions to medicines in general and therefore acted as a proxy measure of participants’ experience of side‐effects in the past. We excluded over 65s due to issues of how representative they are in online surveys. However, over 65s are the heaviest medication consumers,^43^ therefore replicating this work within that population would be of use. It may also be useful for future research to break down ethnicity into more than two categories, to see whether any further differences lie within the “ethnic minority” category. Not only this there is a possibility that risk information also affects the perceived benefits, as well as side‐effects of a medication, as such future work in these field should consider both forms of outcome.

Finally, we suggest that future research should explore ways of reducing participant expectations of side‐effects. Previous research has shown that presenting side‐effect risk with numerical expressions vs verbal labels (eg, 1 in 10 vs common) results in lower expectations.^16^ Another method may be to reframe the numerical risk of side‐effects in terms of the number/proportion of people who remain side‐effect free (eg, 9 in 10 people will not experience).^44^ Alternatively, it may be better to use figurative risk representations that visually display the risk of side‐effects. This has been shown to improve comprehension and accuracy of side‐effect risk interpretation when displayed alongside numerical risk formats.^17^

## 5 CONCLUSION

Members of the public commonly overestimate their own personal likelihood of developing the side‐effects referred to in PILs using the current risk descriptors. This is especially true for women, ethnic minorities, those less educated, those with a household illness and those who have a higher perceived sensitivity to medicines, and negative beliefs about medicines. Interestingly, however, how someone interprets a risk descriptor has little bearing on whether they expect to develop side‐effects themselves. Further research is necessary in order to provide sufficient evidence to reach a conclusion about what should be done regarding verbal descriptors. In the meantime, health‐care professionals should take care to correct any unrealistic expectations patients may have about medication side‐effects to allow patients to make properly informed decisions about medication and to reduce the likelihood of nocebo effects.

## CONFLICTS OF INTEREST

The authors declare no conflicts of interest
