# Supplementary material for: How does the side‐effect information in patient information leaflets influence peoples’ side‐effect expectations? A cross‐sectional national survey of 18‐ to 65‐year‐olds in England
Source: Health Expect. 2017 Jun 15;20(6):1411–20. doi: 10.1111/hex.12584 (PMC5689242; doi:10.1111/hex.12584)
Supplement: Supplementary file 1 [file HEX-20-1411-s001.docx]

**Topline results**

- Results are based on all respondents (1,003) unless otherwise stated.
- Where percentages do not sum to 100, this may be due to respondents being able to select multiple responses, computer rounding or the exclusion of ‘don’t know’/ not stated.
- An asterisk (*) represents a value of less than half or one percent, but greater than zero.
- The response rate for this survey was **9%** (1,003 responses from 11,657 people that the link was emailed to).
- 115 participants ended the survey without completing it (and were not included in the data); 37 people were excluded from the data due to being identified as ‘speeding’ or straightlining’; i.e. completing the survey too quickly to have given genuine, considered answers, or providing identical answers to five or more consecutive questions where this was possible.

**Q1. Please type in your age:**

|  | **%** |
| --- | --- |
| 18-24 | 15 |
| 25-34 | 22 |
| 35-44 | 21 |
| 45-54 | 22 |
| 55-65 | 20 |

**Q2. Which gender do you identify yourself with?**

|  | **%** |
| --- | --- |
| Male | 50 |
| Female | 50 |

**Q3. In which of the following regions do you live?**

|  | **%** |
| --- | --- |
| North East | 5 |
| North West | 13 |
| Yorkshire and Humberside | 10 |
| West Midlands | 10 |
| East Midlands | 8 |
| East of England | 11 |
| South West | 10 |
| South East | 16 |
| London | 17 |

**Q4. Which of the following best describes your employment status?**

|  | **%** |
| --- | --- |
| Working – full time (30 or more hours a week) | 48 |
| Working – part-time (less than 30 hours a week) | 17 |
| Self-employed | 7 |
| Unemployed – looking for a job | 5 |
| Unemployed – not looking for a job/long-term sick or disabled/Housewife/husband/Full-time carer | 12 |
| Retired | 7 |
| Pupil/Student/In full-time education | 5 |
| **NET: Working** | **72** |
| **NET: Not working** | **28** |

**Q7a. A new drug has been developed that has *headache* as one of its listed side effects. The patient information leaflet accompanying the drug states that:**

**“Headache is *uncommon”***

**Imagine 10,000 people take the drug. Out of these people, how many do you think will get a headache?**

|  | **%** |
| --- | --- |
| 0 | * |
| 1 - 100 | 46 |
| 101 - 500 | 22 |
| 501 - 1,000 | 16 |
| 1,001 - 2,500 | 9 |
| 2,501 - 5,000 | 4 |
| 5,001 – 7,500 | 1 |
| 7,501 – 10,000 | 2 |
| **Mean** | **766.1** |

*Base: All in split sample A (506)*

**Q8a. A new drug has been developed that has *headache* as one of its listed side effects. The patient information leaflet accompanying the drug states that:**

**“Headache is *very common”***

**Imagine 10,000 people take the drug. Out of these people, how many do you think will get a headache?**

|  | **%** |
| --- | --- |
| 0 | 0 |
| 1 - 100 | 7 |
| 101 - 500 | 4 |
| 501 - 1,000 | 5 |
| 1,001 - 2,500 | 4 |
| 2,501 - 5,000 | 13 |
| 5,001 – 7,500 | 20 |
| 7,501 – 10,000 | 47 |
| **Mean** | **6249** |

*Base: All in split sample A (506)*

**Q16a. A new drug has been developed that has *nausea* as one of its listed side effects. The patient information leaflet accompanying the drug states that:**

**“Nausea is *rare*”**

**Imagine 10,000 people take the drug. Out of these people, how many do you think will get nausea?**

|  | **%** |
| --- | --- |
| 0 | * |
| 1 - 100 | 60 |
| 101 - 500 | 22 |
| 501 - 1,000 | 12 |
| 1,001 - 2,500 | 3 |
| 2,501 - 5,000 | 2 |
| 5,001 – 7,500 | 0 |
| 7,501 – 10,000 | 1 |
| **Mean** | **455.8** |

*Base: All in split sample A (506)*

**Q18a. A new drug has been developed that has *nausea* as one of its listed side effects. The patient information leaflet accompanying the drug states that:**

**“Nausea is *very rare*”**

**Imagine 10,000 people take the drug. Out of these people, how many do you think will get nausea?**

|  | **%** |
| --- | --- |
| 0 | * |
| 1 - 100 | 70 |
| 101 - 500 | 19 |
| 501 - 1,000 | 8 |
| 1,001 - 2,500 | 1 |
| 2,501 - 5,000 | 1 |
| 5,001 – 7,500 | * |
| 7,501 – 10,000 | 1 |
| **Mean** | **331.9** |

*Base: All in split sample A (506)*

**Q19a. A new drug has been developed that has *nausea* as one of its listed side effects. The patient information leaflet accompanying the drug states that:**

**“Nausea is *common*”**

**Imagine 10,000 people take the drug. Out of these people, how many do you think will get nausea?**

|  | **%** |
| --- | --- |
| 0 | 1 |
| 1 - 100 | 5 |
| 101 - 500 | 4 |
| 501 - 1,000 | 8 |
| 1,001 - 2,500 | 6 |
| 2,501 - 5,000 | 22 |
| 5,001 – 7,500 | 23 |
| 7,501 – 10,000 | 31 |
| **Mean** | **5425.6** |

*Base: All in split sample A (506)*

**Q24a. A new drug has been developed that has *dizziness* as one of its listed side effects. The patient information leaflet accompanying the drug states that:**

**“Dizziness is *very common (may affect more than 1 in 10 people)*”**

**How likely do you think it is that you personally would experience dizziness if you took the drug?**

|  | **%** |
| --- | --- |
| Very likely | 23 |
| Likely | 30 |
| About as likely as not | 23 |
| Unlikely | 18 |
| Very unlikely | 4 |
| Don’t know | 2 |

*Base: All in split sample A (506)*

**Q25a. A new drug has been developed that has *dizziness* as one of its listed side effects. The patient information leaflet accompanying the drug states that:**

**“Dizziness is *common (may affect up to 1 in 10 people)*”**

**How likely do you think it is that you personally would experience dizziness if you took the drug?**

|  | **%** |
| --- | --- |
| Very likely | 11 |
| Likely | 35 |
| About as likely as not | 24 |
| Unlikely | 24 |
| Very unlikely | 4 |
| Don’t know | 2 |

*Base: All in split sample A (506)*

**Q26a. A new drug has been developed that has *dizziness* as one of its listed side effects. The patient information leaflet accompanying the drug states that:**

**“Dizziness is *uncommon (may affect up to 1 in 100 people)*”**

**How likely do you think it is that you personally would experience dizziness if you took the drug?**

|  | **%** |
| --- | --- |
| Very likely | 1 |
| Likely | 6 |
| About as likely as not | 18 |
| Unlikely | 43 |
| Very unlikely | 30 |
| Don’t know | 1 |

*Base: All in split sample A (506)*

**Q27a. A new drug has been developed that has *dizziness* as one of its listed side effects. The patient information leaflet accompanying the drug states that:**

**“Dizziness is *rare (may affect up to 1 in 1000 people)*”**

**How likely do you think it is that you personally would experience dizziness if you took the drug?**

|  | **%** |
| --- | --- |
| Very likely | 1 |
| Likely | 6 |
| About as likely as not | 11 |
| Unlikely | 27 |
| Very unlikely | 55 |
| Don’t know | 1 |

*Base: All in split sample A (506)*

**Q28a. A new drug has been developed that has *dizziness* as one of its listed side effects. The patient information leaflet accompanying the drug states that:**

**“Dizziness is *very rare (may affect up to 1 in 10,000 people)*”**

**How likely do you think it is that you personally would experience dizziness if you took the drug?**

|  | **%** |
| --- | --- |
| Very likely | 1 |
| Likely | 3 |
| About as likely as not | 8 |
| Unlikely | 13 |
| Very unlikely | 74 |
| Don’t know | 2 |

*Base: All in split sample A (506)*

**Q7b. A new drug has been developed that has *difficulty breathing* as one of its listed side effects. The patient information leaflet accompanying the drug states that:**

**“Difficulty breathing is *uncommon*”**

**Imagine 10,000 people take the drug. Out of these people, how many do you think will have difficulty breathing?**

|  | **%** |
| --- | --- |
| 0 | 1 |
| 1 - 100 | 57 |
| 101 - 500 | 21 |
| 501 - 1,000 | 10 |
| 1,001 - 2,500 | 7 |
| 2,501 - 5,000 | 3 |
| 5,001 – 7,500 | * |
| 7,501 – 10,000 | 2 |
| **Mean** | **655.5** |

*Base: All in split sample B (497)*

**Q8b. A new drug has been developed that has *difficulty breathing* as one of its listed side effects. The patient information leaflet accompanying the drug states that:**

**“Difficulty breathing is *very common*”**

**Imagine 10,000 people take the drug. Out of these people, how many do you think will have difficulty breathing?**

|  | **%** |
| --- | --- |
| 0 | 1 |
| 1 - 100 | 12 |
| 101 - 500 | 8 |
| 501 - 1,000 | 8 |
| 1,001 - 2,500 | 6 |
| 2,501 - 5,000 | 13 |
| 5,001 – 7,500 | 18 |
| 7,501 – 10,000 | 35 |
| **Mean** | **5044.8** |

*Base: All in split sample B (497)*

**Q16b. A new drug has been developed that has seizures (fits) as one of its listed side effects. The patient information leaflet accompanying the drug states that:**

**“Seizures (fits) are *rare*”**

**Imagine 10,000 people take the drug. Out of these people, how many do you think will have a seizure (fit)?**

|  | **%** |
| --- | --- |
| 0 | 1 |
| 1 - 100 | 71 |
| 101 - 500 | 16 |
| 501 - 1,000 | 6 |
| 1,001 - 2,500 | 3 |
| 2,501 - 5,000 | 1 |
| 5,001 – 7,500 | 0 |
| 7,501 – 10,000 | 2 |
| **Mean** | **413.2** |

*Base: All in split sample B (497)*

**Q18b. A new drug has been developed that has *seizures (fits)* as one of its listed side effects. The patient information leaflet accompanying the drug states that:**

**“Seizures (fits) are *very rare*”**

**Imagine 10,000 people take the drug. Out of these people, how many do you think will have a seizure (fit)?**

|  | **%** |
| --- | --- |
| 0 | 1 |
| 1 - 100 | 84 |
| 101 - 500 | 9 |
| 501 - 1,000 | 3 |
| 1,001 - 2,500 | 2 |
| 2,501 - 5,000 | 1 |
| 5,001 – 7,500 | 0 |
| 7,501 – 10,000 | 1 |
| **Mean** | **233.3** |

*Base: All in split sample B (497)*

**Q19b. A new drug has been developed that has *seizures (fits)* as one of its listed side effects. The patient information leaflet accompanying the drug states that:**

**“Seizures (fits) are *common*”**

**Imagine 10,000 people take the drug. Out of these people, how many do you think will have a seizure (fit?**

|  | **%** |
| --- | --- |
| 0 | * |
| 1 - 100 | 16 |
| 101 - 500 | 9 |
| 501 - 1,000 | 10 |
| 1,001 - 2,500 | 6 |
| 2,501 - 5,000 | 21 |
| 5,001 – 7,500 | 18 |
| 7,501 – 10,000 | 21 |
| **Mean** | **4171.6** |

*Base: All in split sample B (497)*

**Q24b. A new drug has been developed that has *kidney failure* as one of its listed side effects. The patient information leaflet accompanying the drug states that:**

**“Kidney failure is *very common (may affect more than 1 in 10 people)*”**

**How likely do you think it is that you personally would experience kidney failure if you took the drug?**

|  | **%** |
| --- | --- |
| Very likely | 21 |
| Likely | 30 |
| About as likely as not | 26 |
| Unlikely | 16 |
| Very unlikely | 4 |
| Don’t know | 3 |

*Base: All in split sample B (497)*

**Q25b. A new drug has been developed that has *kidney failure* as one of its listed side effects. The patient information leaflet accompanying the drug states that:**

**“Kidney failure is *common (may affect up to 1 in 10 people)*”**

**How likely do you think it is that you personally would experience kidney failure if you took the drug?**

|  | **%** |
| --- | --- |
| Very likely | 13 |
| Likely | 31 |
| About as likely as not | 27 |
| Unlikely | 23 |
| Very unlikely | 4 |
| Don’t know | 3 |

*Base: All in split sample B (497)*

**Q26b. A new drug has been developed that has *kidney failure* as one of its listed side effects. The patient information leaflet accompanying the drug states that:**

**“Kidney failure is *uncommon (may affect up to 1 in 100 people)*”**

**How likely do you think it is that you personally would experience kidney failure if you took the drug?**

|  | **%** |
| --- | --- |
| Very likely | 2 |
| Likely | 7 |
| About as likely as not | 21 |
| Unlikely | 44 |
| Very unlikely | 24 |
| Don’t know | 3 |

*Base: All in split sample B (497)*

**Q27b. A new drug has been developed that has *kidney failure* as one of its listed side effects. The patient information leaflet accompanying the drug states that:**

**“Kidney failure is *rare (may affect up to 1 in 1000 people)*”**

**How likely do you think it is that you personally would experience kidney failure if you took the drug?**

|  | **%** |
| --- | --- |
| Very likely | 1 |
| Likely | 4 |
| About as likely as not | 14 |
| Unlikely | 34 |
| Very unlikely | 44 |
| Don’t know | 2 |

*Base: All in split sample B (497)*

**Q28b. A new drug has been developed that has *kidney failure* as one of its listed side effects. The patient information leaflet accompanying the drug states that:**

**“Kidney failure is *very rare (may affect up to 1 in 10,000 people)*”**

**How likely do you think it is that you personally would experience kidney failure if you took the drug?**

|  | **%** |
| --- | --- |
| Very likely | 1 |
| Likely | 3 |
| About as likely as not | 10 |
| Unlikely | 14 |
| Very unlikely | 69 |
| Don’t know | 3 |

*Base: All in split sample B (497)*

**Q29. How often, if at all, do you need to have someone help you when you read instructions, pamphlets, or other written material from your doctor or pharmacy?**

|  | **%** |
| --- | --- |
| Never | 64 |
| Rarely | 21 |
| About half the time | 8 |
| Often | 4 |
| Always | 3 |
| Don’t know | 2 |

**Q30. When you take new medications how often, it at all do you read the patient information leaflet accompanying them?**

|  | **%** |
| --- | --- |
| Never | 2 |
| Rarely | 15 |
| About half the time | 19 |
| Often | 23 |
| Always | 39 |
| Don’t know | 2 |

**Q32. The following are things that other people have said about medicines in general. Please indicate the extent to which you agree or disagree with each statement by ticking the appropriate box.**

**There are no right or wrong answers. We are interested in your personal views.**

1. **Doctors use too many medicines**

|  | **%** |
| --- | --- |
| Strongly disagree | 4 |
| Disagree | 22 |
| Uncertain | 36 |
| Agree | 30 |
| Strongly agree | 7 |

1. **People who take medicines should stop their treatment for a while every now and again**

|  | **%** |
| --- | --- |
| Strongly disagree | 15 |
| Disagree | 28 |
| Uncertain | 37 |
| Agree | 17 |
| Strongly agree | 3 |

1. **Most medicines are addictive**

|  | **%** |
| --- | --- |
| Strongly disagree | 16 |
| Disagree | 42 |
| Uncertain | 28 |
| Agree | 12 |
| Strongly agree | 2 |

1. **Natural remedies are safer than medicines**

|  | **%** |
| --- | --- |
| Strongly disagree | 11 |
| Disagree | 25 |
| Uncertain | 45 |
| Agree | 15 |
| Strongly agree | 4 |

1. **Medicines do more harm than good**

|  | **%** |
| --- | --- |
| Strongly disagree | 20 |
| Disagree | 46 |
| Uncertain | 27 |
| Agree | 6 |
| Strongly agree | 1 |

1. **All medicines are poisons**

|  | **%** |
| --- | --- |
| Strongly disagree | 26 |
| Disagree | 37 |
| Uncertain | 23 |
| Agree | 11 |
| Strongly agree | 3 |

1. **Doctors place too much trust on medicines**

|  | **%** |
| --- | --- |
| Strongly disagree | 6 |
| Disagree | 27 |
| Uncertain | 33 |
| Agree | 28 |
| Strongly agree | 7 |

1. **If doctors had more time with patients they would prescribe fewer medicines**

|  | **%** |
| --- | --- |
| Strongly disagree | 3 |
| Disagree | 14 |
| Uncertain | 34 |
| Agree | 39 |
| Strongly agree | 10 |

**Q33. Please indicate how far you agree with each statement by ticking the appropriate box. Be as honest as you can throughout, and try not to let your responses to one question influence your response to other questions. There are no right or wrong answers.**

1. **In uncertain times, I usually expect the best**

|  | **%** |
| --- | --- |
| Strongly disagree | 6 |
| Disagree | 25 |
| Neutral | 42 |
| Agree | 25 |
| Strongly agree | 3 |

1. **It’s easy for me to relax**

|  | **%** |
| --- | --- |
| Strongly disagree | 7 |
| Disagree | 22 |
| Neutral | 26 |
| Agree | 39 |
| Strongly agree | 7 |

1. **If something can go wrong for me, it will**

|  | **%** |
| --- | --- |
| Strongly disagree | 6 |
| Disagree | 32 |
| Neutral | 33 |
| Agree | 23 |
| Strongly agree | 7 |

1. **I’m always optimistic about my future**

|  | **%** |
| --- | --- |
| Strongly disagree | 5 |
| Disagree | 18 |
| Neutral | 33 |
| Agree | 38 |
| Strongly agree | 6 |

1. **I enjoy my friends a lot**

|  | **%** |
| --- | --- |
| Strongly disagree | 1 |
| Disagree | 5 |
| Neutral | 22 |
| Agree | 52 |
| Strongly agree | 20 |

1. **It’s important for me to keep busy**

|  | **%** |
| --- | --- |
| Strongly disagree | 2 |
| Disagree | 9 |
| Neutral | 28 |
| Agree | 49 |
| Strongly agree | 13 |

1. **I hardly ever expect things to go my way**

|  | **%** |
| --- | --- |
| Strongly disagree | 7 |
| Disagree | 32 |
| Neutral | 34 |
| Agree | 21 |
| Strongly agree | 7 |

1. **I don’t get upset too easily**

|  | **%** |
| --- | --- |
| Strongly disagree | 7 |
| Disagree | 22 |
| Neutral | 32 |
| Agree | 33 |
| Strongly agree | 7 |

1. **I rarely count on good things happening to me**

|  | **%** |
| --- | --- |
| Strongly disagree | 7 |
| Disagree | 28 |
| Neutral | 34 |
| Agree | 25 |
| Strongly agree | 6 |

1. **Overall, I expect more good things to happen to me than bad**

|  | **%** |
| --- | --- |
| Strongly disagree | 5 |
| Disagree | 14 |
| Neutral | 34 |
| Agree | 38 |
| Strongly agree | 9 |

**Q34. Please read each of the statements carefully and then select the one which best describes your feelings, over the past six months.**

|  | **%** |
| --- | --- |
| I do not worry about my health | 15 |
| I occasionally worry about my health | 71 |
| I spend much of my time worrying about my health | 12 |
| I spend most of my time worrying about my health | 3 |

**Q35. These are some things other people have said when prescribed medication during regularly scheduled and patient-requested visits. Please indicate to what extent, if at all, you agree or disagree with each statement.**

1. **My body is very sensitive to medicines**

|  | % |
| --- | --- |
| Strongly disagree | 23 |
| Disagree | 45 |
| Uncertain | 22 |
| Agree | 9 |
| Strongly agree | 2 |

1. **My body overreacts to medicines**

|  | % |
| --- | --- |
| Strongly disagree | 27 |
| Disagree | 47 |
| Uncertain | 19 |
| Agree | 5 |
| Strongly agree | 1 |

1. **I usually have stronger reactions to medicines than most people**

|  | % |
| --- | --- |
| Strongly disagree | 27 |
| Disagree | 45 |
| Uncertain | 20 |
| Agree | 7 |
| Strongly agree | 1 |

1. **I have had a bad reaction to medicines in the past**

|  | % |
| --- | --- |
| Strongly disagree | 24 |
| Disagree | 37 |
| Uncertain | 11 |
| Agree | 21 |
| Strongly agree | 6 |

1. **Even very small amounts of medicines can upset my body**

|  | **%** |
| --- | --- |
| Strongly disagree | 26 |
| Disagree | 45 |
| Uncertain | 18 |
| Agree | 9 |
| Strongly agree | 2 |

**Q36. Which of the following ethnic groups would you describe yourself as belonging to?**

|  | **%** |
| --- | --- |
| White – English/Welsh/Scottish/Northern Irish/British | 83 |
| White – Irish | 1 |
| White – Gypsy or Irish Traveller | 1 |
| White – Any other background | 4 |
| Mixed or multiple ethnic groups – White and Black Caribbean | 1 |
| Mixed or multiple ethnic groups – White and Black African | 1 |
| Mixed or multiple ethnic groups – White and Asian | * |
| Mixed or multiple ethnic groups – Any other mixed/multiple ethnic background | 1 |
| Asian or Asian British – Indian | 3 |
| Asian or Asian British – Pakistani | 1 |
| Asian or Asian British – Bangladeshi | 1 |
| Asian or Asian British – Chinese | 1 |
| Asian or Asian British – Any other Asian background | 1 |
| Black, African, Caribbean or Black British – African | 1 |
| Black, African, Caribbean or Black British – Caribbean | 1 |
| Black, African, Caribbean or Black British – Any other Black/African/Caribbean background | 1 |
| Other ethnic group – Arab | * |
| Other ethnic group – Any other ethnic group | * |
| Prefer not to say | 1 |
| **NET: White** | **88** |
| **NET: BME** | **11** |

**Q37. Please state the highest level of education you have achieved.**

|  | **%** |
| --- | --- |
| Left school without qualifications | 5 |
| Secondary education (O-level/GCSE/A-level) | 39 |
| Higher education (BSc/BA/higher qualification) | 56 |
| Prefer not to say | 1 |

**Q38. Do you, or anyone else in your household have any long-standing illness, disability or infirmity?**

|  | **%** |
| --- | --- |
| Yes – myself | 29 |
| Yes – someone else in my household | 13 |
| No | 57 |
| Prefer not to say | 1 |
